# Effects of Government Subsidies on Production and Emissions Reduction Decisions under Carbon Tax Regulation and Consumer Low-Carbon Awareness

**DOI:** 10.3390/ijerph182010959

**Published:** 2021-10-18

**Authors:** Weiling Wang, Yongjian Wang, Xiaoqing Zhang, Dalin Zhang

**Affiliations:** 1 School of Law, Southeast University, Nanjing 211189, China; sandandefeng@126.com; 2 Business School, Jiangsu Normal University, Xuzhou 221116, China; xqzhang22@163.com; 3 Department of Computer Science, Aalborg University, 9220 Aalborg Øst, Denmark

**Keywords:** production decision, emissions reduction decision, government subsidies, carbon tax, low-carbon awareness

## Abstract

To promote low-carbon production, the government simultaneously provides some subsidies under carbon tax regulations. Two government subsidies are widely adopted: one is based on emissions reduction quantity and the other is based on emissions reduction investment cost. Additionally, consumer low-carbon awareness has also been enhanced. Considering the aforementioned circumstances, this paper investigates the effects of different government subsidies on production and emissions reduction decisions under a carbon tax regulation by formulating three decision-making optimization models. The results show that (1) although the carbon tax regulation cannot guarantee further improvement of emissions reduction levels, government subsidies could make the corresponding conditions of improving emissions reduction investments wider; (2) a heavy carbon tax or stronger consumer low-carbon awareness would make the positive effect of government subsidies more apparent; and (3) subsidy policies may also be selected by the government from different perspectives, such as manufacturer development, consumer surplus, environmental damage and social welfare. Especially, from the perspective of maximizing social welfare, investment cost (IC) subsidy is not always advantageous, while emissions reduction (ER) subsidy can always bring higher social welfare compared with the case under no government subsidy.

## 1. Introduction

Climate change has become a focus issue and has attracted extensive attention from the international community. To control carbon emissions, many countries have promulgated several policies and regulations, such as mandatory carbon emissions capacity, carbon emissions cap and trade, carbon tax, and low-carbon offset [1,2]. Among them, carbon tax regulation is served as an effective policy to reduce carbon emissions [3,4], and has been implemented in many countries, such as Sweden, Ireland and the U.S. [5,6]. However, studies have shown that the carbon tax regulation has limited effect in promoting low-carbon emissions reduction activities [7,8], and also results in increased operational costs, loss of profits for manufacturers and slow economic growth to some degree [9,10,11,12]. Thus, in practice, national governments provide some preferential policies to improve manufacturers’ enthusiasm of emissions reduction, such as subsidies, tax incentives, government priority procurement, etc. The government subsidy is identified as one of the most common and effective policies [13,14,15], and two of which are widely used, including a subsidy based on emissions reduction quantity (ER subsidy) and the other one based on emissions reduction investment cost (IC subsidy) [16]. For instance, since 2007, the ‘Energy Act’ and ‘Economic Stability Act’ issued by the U.S. government announced that it would subsidize the research and development (R&D), production and consumption of new energy vehicles. In Japan, the government invests 57 billion yen in renewable energy technology R&D every year through the ‘New Sunshine Program’, and encourages enterprises to actively conduct energy conservation and emissions reduction by direct subsidies. For example, enterprises can receive a subsidy of 50% of the investment cost when introducing renewable energy technologies [17]. Additionally, the ‘Interim Measures of the Subsidies Administration for Energy Conservation and Emission Reduction’ introduced in 2015 and revised in 2020 by the Chinese government, clearly states that subsidies could be allocated by investment cost, energy conservation and emissions reduction effect, etc. A typical example is Shenzhen government’s subsidies of 40% of investment cost when enterprises purchase the water-based coating equipment that can reduce volatile organic compounds emission [18]. Another example of improving emissions reduction activities of volatile organic compounds (VOCs) is that Shanghai government introduced the ‘Special Support Measures for Deepen Governance Projects of Volatile Organic Compounds in Key Industries’ in August 2021, in which grading government subsidies are provided based on emissions reduction quantity [19]. However, in the actual implementation process, different government subsidies would bring differentiated emissions reduction promotion effects. Thus, how the government subsidies affect manufacturers’ low-carbon production and how to choose the appropriate subsidy policy to truly maximize economic and environmental benefits are worthy of discussion.

In addition, more and more consumers have been environmentally conscious and have a stronger willingness to purchase low-carbon products [6,8,20]. A global survey conducted by Accenture reveals that more than 80% of consumers pay attention to the environmental property of products when purchasing them [15]. Another survey demonstrates that 70–80% of local consumers prefer to purchase environmentally-friendly household electronic and electrical equipment in Ningbo (Zhejiang Province, China) [21]. The increasing consumers’ low-carbon awareness becomes a market-driven factor that encourages manufacturers to launch emissions reduction activities [22].

Facing carbon regulations, government subsidies and consumers’ low-carbon awareness, emissions reduction has been incorporated into operational planning by manufacturers [23,24,25]. For instance, Gree, Haier and other manufacturers have also incessantly launched low-carbon products to enhance environmental image and gain competitive advantages as well as have obtained some achievements in environmental protection [26]. Thus, manufacturers need to explore optimal emissions reduction level and the corresponding production decision, comprehensively considering all of the relevant factors. According to manufacturers’ decision feedback, the government determines which subsidy policy should be implemented to better promote emissions reduction under a carbon tax regulation. In such a context, this study seeks to address the following questions:

(1) How do different government subsidies influence manufacturers’ production and emissions reduction decisions under the carbon tax regulation?
(2) What is the subsidy effect of different government subsidies under the identical subsidy rate? How do carbon tax and consumer low-carbon awareness influence the effect of different government subsidies?
(3) How does the government determine which subsidy policy should be adopted?

To address the above issues in this paper, with consideration of carbon tax regulation and consumer low-carbon awareness, integrated optimization models of production and emissions reduction decisions are formulated under two government subsidy policies (e.g., ER subsidy and IC subsidy). Meanwhile, some theoretical analysis and numerical analysis has been conducted to analyze and illustrate effects of carbon tax regulation, consumer low-carbon awareness, emissions reduction cost coefficient and government subsidies on the manufacturer’s operational decisions. Conclusions and managerial insights have also been derived from the analysis.

The remainder of this paper is organized as follows. Section 2 is devoted to a review of the related literature. The problem is described and the corresponding assumptions are provided in Section 3. Three profit maximization models under different government subsidies are formulated and solved in Section 4. Section 5 presents some theoretical analysis and some numerical analysis is provided in Section 6. Finally, this study ends with conclusions and managerial insights in Section 7.

## 2. Literature Review

This study is closely related to two main streams of literature: (1) the literature addressing enterprise/supply chain decisions under carbon tax regulations, and (2) the literature exploring effects of government subsidies on enterprise/supply chain decisions.

In the first stream of literature, enterprise/supply chain decisions under carbon tax regulations have received considerable research attention, some of which only study operational decisions [5,24,27,28,29]. Some papers involve integrated decisions of operational and emissions reduction. For instance, under the modified wholesale price and modified cost-sharing contract, Yu and Han [30] probed impacts of a carbon tax on manufacturers’ abatement effort level and retailers’ retail price in a two-echelon supply chain. Yang and Chen [31] studied effects of a revenue-sharing scheme and cost-sharing scheme provided by a retailer on a manufacturer’s emissions abatement and profitability of both enterprises under consumer environmental awareness and carbon tax. Considering the carbon tax and takeback legislation, Ding et al. [4] explored remanufacturing and emissions reduction strategies under monopolistic and competitive scenarios. Alegoz et al. [32] compared and analyzed production and abatement level decisions in a pure manufacturing system and a hybrid manufacturing/remanufacturing system under the carbon tax regulation. Huang and Zhang [33] investigated optimal pricing and emissions reduction decisions of a two-echelon supply chain under a carbon tax and different power structures. It can be found that the carbon tax regulation could promote manufacturers’ emissions reduction but is not beneficial to their profits.

Moreover, effects of government subsidies on green technology investment or new energy development have been widely concerned by academic communities, as exemplified by Lin and Jiang [34] and Cohen et al. [35]. This paper mainly focuses on the literature of enterprise/supply chain decisions under government subsidies. Some papers concentrate on the price subsidy based on production quantity, such as Wang et al. [36], Hussain et al. [37], Yang et al. [12] and Meng et al. [26]. However, some papers involved in R&D subsidy are mainly based on the energy-saving level (or emissions reduction level) of the products regardless of the related investment cost, such as Yu et al. [38], Xue et al. [39] and Lou et al. [40]. To the best of our knowledge, there is scant literature involved in a single government subsidy policy between IC subsidy and ER subsidy to study enterprise/supply chain decisions. The related research mostly focus on comparison of different government subsidies. For instance, Wang et al. [41] showed that the IC subsidy could reasonably control the quantity of remanufacturers and maintain remanufacturing industry scale and stability when the remanufacturing industry develops to a certain extent by comparing IC subsidy with initial subsidy, recycling subsidy and production subsidy. Guo et al. [42] mainly investigated effects of government subsidies based on greenness efforts cost and production quantity on social welfare and profits of supply chain members, and found that the subsidy policy which is more efficient depends on manufacturers’ marginal profits and consumers’ price sensitivity. Considering consumers’ green preference, Wen et al. [43] indicated that the IC subsidy would bring higher product green degree, total profit of the manufacturer and retailer compared with subsidies based on production cost and green degree. Song et al. [44] explored how to promote sustainable innovation in university-industry collaboration considering different investment subsidies for universities and research institutes and companies. Furthermore, concentrated on two types of subsidy policies introduced in our paper, He et al. [45] discussed effects of different government subsidies on supply chain operational decisions based on the assumption of equivalent total subsidies under specific emissions reduction actions. Li et al. [46] studied green decisions of a two-echelon supply chain under cap-and-trade mechanism based on a fixed green technology investment cost and concluded the conditions or circumstances under which a particular subsidy policy is more effective. In sum, the existing literature rarely focuses on the comparison of ER subsidy and IC subsidy, and does not consider the carbon tax regulation that affects the amount of government subsidy under ER subsidy.

Regardless, both carbon tax regulation and government subsidy policies are effective ways to curb carbon emissions. Considerable attention has been devoted to comparing carbon tax regulations with government subsidy policies, as exemplified by Galinato and Yoder [47], Zhao et al. [48], Cao et al. [8] and Zhou et al. [6]. In some countries or regions, these two types of policies are adopted simultaneously, such as in the U.S. and Korea [5,42,49]. Furthermore, some studies have shown that the combination of carbon tax regulations and government subsidies is beneficial to economic development while achieving emissions reduction targets [49,50]. Nevertheless, there are few papers focusing on the comparison of different government subsidies based on the integration of carbon tax regulations. Although Shu et al. [10] conducted a comparative analysis about effects of different subsidy policies on pricing and production decisions under a carbon tax regulation, it considers price subsidy for remanufactured products and carbon tax rebate and no emissions reduction issue.

Therefore, given this gap in research, this paper focuses on ER subsidy and IC subsidy and explores their effects on integrated decisions of production and emissions reduction under a carbon tax regulation. Unlike the existing literature, the amount of government subsidy under ER subsidy is closely related to carbon tax rate provided by the government. Consequently, we emphatically investigate the influence of carbon tax on operational decisions, the environment and the effect of the above two types of subsidy policies besides analyzing the influence of the consumer low-carbon awareness. Meanwhile, this paper further discusses how to determine which particular subsidy policy will be more effective considering different circumstances and perspectives. Finally, analysis results provide some managerial insights for manufacturers and government.

## 3. Problem Description and Assumptions

### 3.1. Problem Description and Symbol Instruction

This study considers a monopolistic manufacturer selling low-carbon products directly. To promote the development of low-carbon economy, a carbon tax regulation is implemented by the government. Meanwhile, two government subsidy policies (e.g., ER subsidy and IC subsidy) are provided. The manufacturer initially determines on emissions reduction level and then on optimal production quantity of low-carbon products to maximize its profit under the given tax and subsidy rates. To clarify our model, the parameters and variables involved in the models are shown in Table 1.

### 3.2. Problem Description and Symbol Instruction

To better understand our model, the key assumptions are shown as follows:

**Assumption** **1.** *Referring to Cao et al. [51] and Yang et al. [12], the government acts as the leader and needs to determine tax price, specific subsidy policy and subsidy rate. The manufacturer is the follower and committed to maximizing its profit, and determines emissions reduction level and production quantity of low-carbon products successively.*

**Assumption** **2.** *Following the example of earlier studies [30,51,52], the market demand of low-carbon products is sensitive to product sales price and emissions reduction level. Moreover, it should be noted that manufacturing quantity q is equal to the market demand. Then, the demand function of low-carbon products can be expressed as q = α-βp+γτ. Thus, the inverse function can be derived as* $p=\frac{\alpha +\gamma \tau -q}{\beta }$.

**Assumption** **3.** *Since the government sets a carbon tax rate of low-carbon products t_0_, the corresponding emissions costs are t_0_e. Similar to Zhou et al. [53], Ding et al. [4] and Wang and Wang [54], other manufacturing costs in our model are ignored, which is more helpful for us to reveal the central issues.*

**Assumption** **4.** *The manufacturer’s emissions reduction activity can be regarded as one-time investments. The higher the emissions reduction level is, the higher the emissions reduction cost. Similar to Jones and Mendelson [55], Wei et al. [56] and Ding et al. [4], the emissions reduction cost is assumed to be a quadratic function* $\frac{1}{2}k\tau^{2}$, *which follows the law of diminishing returns on investment*.

**Assumption** **5.** *To encourage manufacturers to launch low-carbon production, two government subsidy policies are provided. (1) ER subsidy based on emissions reduction quantity: the government subsidizes manufacturers according to carbon taxes payable of emissions reductions quantity. Here, t_0_ and η respectively represent carbon tax rate and subsidy rate, then government subsidy expenditure is ηt_0_·τeq. (2) IC subsidy based on emissions reduction investment cost: the government subsidizes manufacturers according to one-time emissions reduction investments, similar assumption can be found in Wang et al. [41], Guo et al. [42] and Wen et al. [43]. Then, combined with Assumption 4, the government subsidy expenditure is* $\frac{\eta }{2}k\tau_{2}^{2}$*when the subsidy rate is alsoη*.

## 4. Manufacturer’s Decision-Making Models

In order to analyze effects of different government subsidies on emissions reduction and production decisions under a carbon tax regulation, three decision-making models are formulated in this section; one is the model without government subsidy, one is the model with ER subsidy and another one is the model with IC subsidy. The proofs of all propositions are provided in Appendix A.

### 4.1. Decision-Making Model under No Government Subsidy

In this subsection, the government levies a carbon tax on the manufacturer and does not provide any subsidy policy. Then, according to the problem description mentioned above, the manufacturer’s profit function is:

(1) $$\pi_{0}=[\frac{\alpha +\gamma \tau_{0}-q_{0}}{\beta }-t_{0}e(1-\tau_{0})]q_{0}-\frac{1}{2}k\tau_{0}^{2}$$

where the first and second terms represent net sales revenue of low-carbon products, and the last term denotes the manufacturer’s emissions reduction investment cost.

**Proposition** **1.** *Under no government subsidy, total profit*$\;\pi_{0}$*is jointly concave with respect to*$\;\tau_{0}$*and*$\;q_{0}$, *and optimal value s*$\;\tau_{0}^{*}$,$\;q_{0}^{*}$,$\;\pi_{0}^{*}$,$\;E_{0}$*are as follows:*$\;\tau_{0}^{*}=\frac{\left(\alpha -\beta t_{0}e)(\gamma +\beta t_{0}e\right)}{2k\beta -{\left(\gamma +\beta t_{0}e\right)}^{2}}$*,*$\;q_{0}^{*}=\frac{k\beta (\alpha -\beta t_{0}e)}{2k\beta -{\left(\gamma +\beta t_{0}e\right)}^{2}}$*,*$\;\pi_{0}^{*}=\frac{k{\left(\alpha -\beta t_{0}e\right)}^{2}}{4k\beta -2{\left(\gamma +\beta t_{0}e\right)}^{2}}$*,*$\;E_{0}=\frac{k\beta e(\alpha -\beta t_{0}e)[2k\beta -(\alpha +\gamma )(\gamma +\beta t_{0}e)]}{{\left[2k\beta -{\left(\gamma +\beta t_{0}e\right)}^{2}\right]}^{2}}$*, in which*$\;\alpha -\beta t_{0}e>0$*,*$\;2k\beta -(\alpha +\gamma )(\gamma +\beta t_{0}e)>0$.

### 4.2. Decision-Making Model under ER Subsidy

In this subsection, the government levies a carbon tax and simultaneously subsidizes the manufacturer based on carbon taxes payable of emissions reductions quantity, and the corresponding subsidy rate is *η*. The amount of government subsidy can be expressed as *ηt*_0_·*τ*_1_*eq*_1_. Then, according to the problem description mentioned above, the manufacturer’s profit function is:

(2) $$\pi_{1}=[\frac{\alpha +\gamma \tau_{1}-q_{1}}{\beta }-t_{0}(1-\tau_{1})e]q_{1}+\eta t_{0}\cdot \tau_{1}eq_{1}-\frac{1}{2}k\tau_{1}{}^{2}$$

**Proposition** **2.** *Under the ER subsidy, total profit*$\;\pi_{1}$*is jointly concave with respect to*$\;\tau_{1}$*and*$q_{1}$*, and optimal values*$\tau_{1}^{*}$,$q_{1}^{*}$,$\pi_{1}^{*}$,$\;E_{1}$*are as follows:*$\tau_{1}^{*}=\frac{\left(\alpha -\beta t_{0}e)[\gamma +\beta t_{0}e(1+\eta )\right]}{2k\beta -{\left[\gamma +\beta t_{0}e(1+\eta )\right]}^{2}}$*,*$q_{1}^{*}=\frac{k\beta (\alpha -\beta t_{0}e)}{2k\beta -{\left[\gamma +\beta t_{0}e(1+\eta )\right]}^{2}}$*,*$\pi_{1}^{*}=\frac{k{\left(\alpha -\beta t_{0}e\right)}^{2}}{4k\beta -2{\left[\gamma +\beta t_{0}e(1+\eta )\right]}^{2}}$*,*$E_{1}=\frac{k\beta e(\alpha -\beta t_{0}e)\{2k\beta -[\alpha (1+\eta )+\gamma ][\gamma +\beta t_{0}e(1+\eta )]\}}{{\left\{2k\beta -{\left[\gamma +\beta t_{0}e(1+\eta )\right]}^{2}\right\}}^{2}}$*,in which*$\alpha -\beta t_{0}e>0$*and*$2k\beta -[\alpha (1+\eta )+\gamma ][\gamma +\beta t_{0}e(1+\eta )]>0$.

### 4.3. Decision-Making Model under IC Subsidy

In this subsection, the government levies a carbon tax and simultaneously subsidizes the manufacturer based on one-time emissions reduction investment cost, and the corresponding subsidy rate is also *η*. The amount of government subsidy can be expressed as $\frac{\eta }{2}k\tau_{2}^{2}$. Then, according to the problem description mentioned above, the manufacturer’s profit function is:

(3) $$\pi_{2}=[\frac{\alpha +\gamma \tau_{2}-q_{2}}{\beta }-t_{0}e(1-\tau_{2})]q_{2}-(1-\eta )\frac{1}{2}k\tau_{2}^{2}$$

**Proposition** **3.** *Under the IC subsidy, total profit*$\pi_{2}$*is jointly concave with respect to*$\tau_{2}$*and*$q_{2}$*, and optimal values*$\tau_{2}^{*}$,$q_{2}^{*}$,$\pi_{2}^{*}$,$E_{2}$*are as follows:*$\tau_{2}^{*}=\frac{\left(\alpha -\beta t_{0}e)(\gamma +\beta t_{0}e\right)}{2k\beta (1-\eta )-{\left(\gamma +\beta t_{0}e\right)}^{2}}$*,*$q_{2}^{*}=\frac{k\beta (1-\eta )(\alpha -\beta t_{0}e)}{2k\beta (1-\eta )-{\left(\gamma +\beta t_{0}e\right)}^{2}}$*,*$\pi_{2}^{*}=\frac{k(1-\eta ){\left(\alpha -\beta t_{0}e\right)}^{2}}{4k\beta (1-\eta )-2{\left(\gamma +\beta t_{0}e\right)}^{2}}$*,*$E_{2}=\frac{k\beta e(\alpha -\beta t_{0}e)(1-\eta )[2k\beta (1-\eta )-(\alpha +\gamma )(\gamma +\beta t_{0}e)]}{{\left[2k\beta (1-\eta )-{\left(\gamma +\beta t_{0}e\right)}^{2}\right]}^{2}}$*,in which*$\alpha -\beta t_{0}e>0$*and*$2k\beta (1-\eta )-(\alpha +\gamma )(\gamma +\beta t_{0}e)>0$.

## 5. Result Analysis and Discussion

This section analyzes the equilibrium results in the above three models and conducts a comparative analysis on the effects of different government subsidy policies on emissions reduction level, manufacturing quantity and total profit. The proofs of all corollaries are also provided in Appendix A.

**Corollary** **1.** *Under a carbon tax regulation, the manufacturer’s optimal manufacturing quantity, total profit and carbon emissions are respectively* $q_{n}^{*}$and$\pi_{n}^{*}$when emissions reduction investment is not considered. Then, effects of the emissions reduction are as follows: (1)$q_{0}^{*}>q_{n}^{*}$, $q_{1}^{*}>q_{n}^{*}$, $q_{2}^{*}>q_{n}^{*}$;(2)$\pi_{0}^{*}>\pi_{n}^{*}$, $\pi_{1}^{*}>\pi_{n}^{*}$, $\pi_{2}^{*}>\pi_{n}^{*}$.

Corollary 1 implies that, under a carbon tax regulation, the manufacturer can always improve its manufacturing quantity and total profit through launching emissions reduction activities for the above three cases. Thus, although the carbon tax regulation would increase emissions cost, it can effectively promote manufacturers’ low-carbon production and then achieve the higher profits.

**Corollary** **2.** *When the manufacturer launches low-carbon production, effects of the carbon tax regulation on emissions reduction decisions are as follows: (1) If* $t_{0}<\frac{\alpha -\gamma }{2\beta e}$, *then*$\frac{\partial \tau_{0}^{*}}{\partial t_{0}}>0$; *if*$t_{0}>\frac{\alpha -\gamma }{2\beta e}$, *only when*$k<\frac{(\alpha +\gamma ){\left(\gamma +\beta t_{0}e\right)}^{2}}{2\beta (\gamma -\alpha +2\beta t_{0}e)}$, $\frac{\partial \tau_{0}^{*}}{\partial t_{0}}>0$, *otherwise,*$\frac{\partial \tau_{0}^{*}}{\partial t_{0}}<0$. *(2) If*$t_{0}<\frac{\alpha (1+\eta )-\gamma }{2\beta e(1+\eta )}$, *then*$\frac{\partial \tau_{1}^{*}}{\partial t_{0}}>0$; *if*$t_{0}>\frac{\alpha (1+\eta )-\gamma }{2\beta e(1+\eta )}$, *only when*$k<\frac{[\alpha (1+\eta )+\gamma ]{\left[\gamma +\beta t_{0}e(1+\eta )\right]}^{2}}{2\beta [\gamma -\alpha +2\beta t_{0}e(1+\eta )]}$, $\frac{\partial \tau_{1}^{*}}{\partial t_{0}}>0$, *otherwise,*$\frac{\partial \tau_{1}^{*}}{\partial t_{0}}<0$. *(3) If*$t_{0}<\frac{\alpha -\gamma }{2\beta e}$, *then*$\frac{\partial \tau_{2}^{*}}{\partial t_{0}}>0$; *if*$t_{0}>\frac{\alpha -\gamma }{2\beta e}$, *only when*$k<\frac{[\alpha (1+\eta )+\gamma ]{\left[\gamma +\beta t_{0}e(1+\eta )\right]}^{2}}{2\beta [\gamma -\alpha +2\beta t_{0}e(1+\eta )]}$, $\frac{\partial \tau_{2}^{*}}{\partial t_{0}}>0$, *otherwise,*$\frac{\partial \tau_{2}^{*}}{\partial t_{0}}<0$.

Corollary 2 indicates that, under a carbon tax regulation, the rising tax rate can always encourage the manufacturer to improve emissions reduction levels when it is lower than a certain critical value under each case. However, if the tax rate is higher than the corresponding critical value, the rising tax rate would improve emissions reduction levels only when emissions reduction investment cost is low. Thus, for the government, although a carbon tax regulation can effectively promote manufacturers to launch emissions reduction activities, it is essential to be targeted to raise or reduce the tax rate to further improve manufacturers’ emissions reduction levels while considering emissions reduction technology or investment cost.

**Corollary** **3.** *When the manufacturer launches low-carbon production, effects of the carbon tax regulation on manufacturing quantities and total profits are as follows: (1) If*$k<\frac{\left(2\alpha +\gamma -\beta t_{0}e)(\gamma +\beta t_{0}e\right)}{2\beta }$, *then*$\frac{\partial q_{0}^{*}}{\partial t_{0}}>0$*and*$\frac{\partial \pi_{0}^{*}}{\partial t_{0}}<0$; *if*$k>\frac{\left(2\alpha +\gamma -\beta t_{0}e)(\gamma +\beta t_{0}e\right)}{2\beta }$, *then*$\frac{\partial q_{0}^{*}}{\partial t_{0}}<0$*and*$\frac{\partial \pi_{0}^{*}}{\partial t_{0}}<0$. *(2) If*$k<\frac{\left[2\alpha (1+\eta )+\gamma -\beta t_{0}e(1+\eta )][\gamma +\beta t_{0}e(1+\eta )\right]}{2\beta }$, *then*$\frac{\partial q_{1}^{*}}{\partial t_{0}}>0$*and*$\frac{\partial \pi_{1}^{*}}{\partial t_{0}}<0$; *if*$k>\frac{\left[2\alpha (1+\eta )+\gamma -\beta t_{0}e(1+\eta )][\gamma +\beta t_{0}e(1+\eta )\right]}{2\beta }$, *then*$\frac{\partial q_{1}^{*}}{\partial t_{0}}<0$*and*$\frac{\partial \pi_{1}^{*}}{\partial t_{0}}<0$. *(3) If*$k<\frac{\left(2\alpha +\gamma -\beta t_{0}e)(\gamma +\beta t_{0}e\right)}{2\beta (1-\eta )}$, *then*$\frac{\partial q_{2}^{*}}{\partial t_{0}}>0$*and*$\frac{\partial \pi_{2}^{*}}{\partial t_{0}}<0$; *if*$k>\frac{\left(2\alpha +\gamma -\beta t_{0}e)(\gamma +\beta t_{0}e\right)}{2\beta (1-\eta )}$, *then*$\frac{\partial q_{2}^{*}}{\partial t_{0}}<0$*and*$\frac{\partial \pi_{2}^{*}}{\partial t_{0}}<0$.

It can be found from Corollary 3 that a carbon tax regulation always decreases the manufacturer’s total profit for the above three cases. However, whether the carbon tax regulation could improve manufacturing quantity of low-carbon products mainly depends on emissions reduction investment cost. When the emissions reduction cost coefficient is higher than a certain critical value, the manufacturer’s manufacturing quantity will be decreasing with the increase of tax rate under each case. Consequently, the rising emissions cost forces the manufacturer to reduce manufacturing quantity. Then, it is necessary for the government to levy a lower carbon tax on manufacturers with underdeveloped emissions reduction technologies or higher emission reduction costs to properly promote emissions reduction and simultaneously protect low-carbon production activities.

**Corollary** **4.** *Under a carbon tax regulation, effects of the low-carbon sensitivity coefficient and emissions reduction cost coefficient on optimal emissions reduction decisions, manufacturing quantities and total profits are as follows: (1)* $\frac{\partial \tau_{0}^{*}}{\partial \gamma }>0$, $\frac{\partial \tau_{1}^{*}}{\partial \gamma }>0$, $\frac{\partial \tau_{2}^{*}}{\partial \gamma }>0$*and*$\frac{\partial \tau_{0}^{*}}{\partial k}<0$, $\frac{\partial \tau_{1}^{*}}{\partial k}<0$, $\frac{\partial \tau_{2}^{*}}{\partial k}<0$; *(2)*$\frac{\partial q_{0}^{*}}{\partial \gamma }>0$, $\frac{\partial q_{1}^{*}}{\partial \gamma }>0$, $\frac{\partial q_{2}^{*}}{\partial \gamma }>0$*and*$\frac{\partial q_{0}^{*}}{\partial k}<0$, $\frac{\partial q_{1}^{*}}{\partial k}<0$, $\frac{\partial q_{2}^{*}}{\partial k}<0$; *(3)*$\frac{\partial \pi_{0}^{*}}{\partial \gamma }>0$, $\frac{\partial \pi_{1}^{*}}{\partial \gamma }>0$, $\frac{\partial \pi_{2}^{*}}{\partial \gamma }>0$*and*$\frac{\partial \pi_{0}^{*}}{\partial k}<0$, $\frac{\partial \pi_{1}^{*}}{\partial k}<0$, $\frac{\partial \pi_{2}^{*}}{\partial k}<0$.

Corollary 4 shows that the rising low-carbon sensitivity coefficient or the declining emissions reduction cost coefficient can always improve the manufacturer’s emissions reduction levels, manufacturing quantities and total profits for the above three cases. Thus, it is significant for the government and manufacturers to positively propagandize low-carbon products to enhance consumers’ low-carbon preference and loyalty. Meanwhile, it is essential for manufacturers to independently develop or introduce efficient and low-cost emissions reduction technologies.

**Corollary** **5.** *Under a carbon tax regulation, compared with the case under no government subsidy, effects of government subsidies on optimal emissions reduction decisions, manufacturing quantities and total profits are as follows: (1)* $\tau_{1}^{*}>\tau_{0}^{*}$, $\tau_{2}^{*}>\tau_{0}^{*}$; *(2)*$q_{1}^{*}>q_{0}^{*}$, $q_{2}^{*}>q_{0}^{*}$; *(3)*$\pi_{1}^{*}>\pi_{0}^{*}$and$\pi_{2}^{*}>\pi_{0}^{*}$.

Corollary 5 illustrates that both ER subsidy and IC subsidy could improve the manufacturer’s emissions reduction levels, manufacturing quantities and total profits. This also indicates that the manufacturer will increase emissions reduction investments and production quantities of low-carbon products under the above two subsidy policies. Accordingly, higher sales revenue increments and emissions cost decrements bring higher total profits to the manufacturer. Therefore, a reasonable government subsidy policy is necessary to provide to manufacturers who launch emissions reduction activities.

**Corollary** **6.** *Under a carbon tax regulation, effects of different government subsidies on optimal emissions reduction decisions are as follows: if* $t_{0}e(\gamma +\beta t_{0}e)[\gamma +\beta t_{0}e(1+\eta )]-2k(\gamma +\eta \beta t_{0}e)>0$, *then*$\tau_{1}^{*}>\tau_{2}^{*}$; *otherwise,*$\tau_{1}^{*}<\tau_{2}^{*}$.

**Corollary** **7.** *Under a carbon tax regulation, effects of different government subsidies on optimal manufacturing quantity and total profit are as follows: if* $(1-\eta ){\left[\gamma +\beta t_{0}e(1+\eta )\right]}^{2}-{\left(\gamma +\beta t_{0}e\right)}^{2}>0$, *then*$q_{1}^{*}>q_{2}^{*}$and$\pi_{1}^{*}>\pi_{2}^{*}$; *otherwise,*$q_{1}^{*}<q_{2}^{*}$and$\pi_{1}^{*}<\pi_{2}^{*}$.

It can be perceived from Corollary 6 and Corollary 7 that the emissions reduction level under IC subsidy is higher compared with that under ER subsidy for manufacturers with higher emissions reduction cost. Conversely, when the emissions reduction cost is effectively controlled by manufacturers, ER subsidy could better improve emissions reduction activities. However, which government subsidy policy is more beneficial to production activities and the total profit are independent of emissions reduction cost. In addition, which government subsidy is more beneficial to emissions reduction investment, production activities and total profit also depend on carbon tax rate, low-carbon sensitivity coefficient and subsidy rate. Specific effects will be analyzed by numerical analysis in the next section.

## 6. Numerical Analysis and Results Discussion

Referring to Ding et al. [4], Wang and Wang [54] and combining with the practices, this section provides numerical examples to illustrate the above analytical results, and further discusses effects of critical parameters such as carbon tax rate, low-carbon sensitivity coefficient and government subsidy rate on emissions reduction quantity, social welfare. Specifically, the ranges for each parameter are as follows: *α >* 0, *β* > 0, *t*_0_ *>* 0, *k >* 0, *e >* 0, *μ >* 0, *γ >* 0 and *η* ϵ (0, 1).

### 6.1. Effects of Carbon Tax Regulation

**Figure 1:**
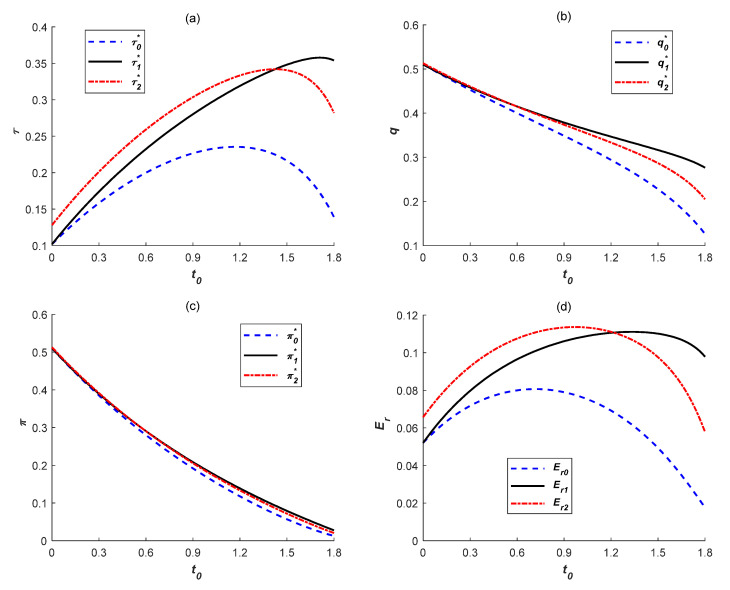
Effects of carbon tax rate on (**a**) emissions reduction levels, (**b**) manufacturing quantities, (**c**) total profits and (**d**) emissions reduction quantities.

To explore the effects of carbon tax rate on emissions reduction levels, manufacturing quantities, total profits and emissions reduction quantities in the above three cases, the basic parameters used for this numerical analysis are shown in detail as follows. The potential demand of low-carbon products is 1 (*α* = 1). The product price sensitivity coefficient is 0.5 (*β* = 0.5), which represents a change in potential demand caused by a change in price for low-carbon products. In addition, the low-carbon sensitivity coefficient is 0.2 (*γ* = 0.2), which denotes consumers’ preference degree for low-carbon products. The emissions reduction cost coefficient is 2 (*k* = 2), which determines one-time emissions reduction investment cost. Furthermore, the emissions quantity of unit low-carbon product is 1 (*e* = 1) without emissions reduction activities. Finally, the subsidy rate was set as 0.2 (*η* = 0.2) and the results are shown in Figure 1.

As shown in Figure 1a, the rising tax rate *t*_0_ would not always promote the manufacturer to improve emissions reduction levels in the above three cases. In each case, the emissions reduction cost will play a more critical role in determining emissions reduction level as *t*_0_ increases. When other parameters are constant, the rising *t*_0_ would lead to the decline in the critical value of emissions reduction cost coefficient forcing the manufacturer to begin to reduce the emissions reduction level. Therefore, with the increase of *t*_0_, emissions reduction levels initially increase and then decrease, which is consistent with Corollary 2. However, government subsidies create wider conditions of *t*_0_ in improving emissions reduction investments, especially under ER subsidy. In this way, the emissions reduction cost coefficient is always higher than the certain critical value of *k* forcing the manufacturer to begin to decrease low-carbon products in each case. Consequently, because of the higher emissions reduction cost, the manufacturer’s optimal manufacturing quantities and total profits are declining with the increase of *t*_0_ as shown in Figure 1b,c, which is also consistent with Corollary 3. Accordingly, as *t*_0_ increases, the manufacturer’s emissions reduction quantities also initially increase and then decrease since manufacturing quantities are decreasing and growth rates of emissions reduction levels are declining, or even negative. This implies that although a carbon tax regulation can promote manufacturers’ emissions reduction levels to some degree, a higher tax rate would weaken manufacturers’ tolerance of emissions reduction cost, thereby severely restraining their production activities and total profits and discouraging manufacturers’ enthusiasm to further improve emissions reduction levels.

In addition, all variable values are higher under both government subsidies compared with those under no government subsidy. Furthermore, generally speaking, the difference in each value is increasing as *t*_0_ increases, as shown in Figure 1. This indicates that a reasonable government subsidy policy is necessary to provide to manufacturers who launch emissions reduction activities, which is consistent with Corollary 5. Meanwhile, in a heavy carbon tax circumstance, the positive effect of government subsidies would be more apparent. Finally, as shown in Figure 1, each variable value under ER subsidy is gradually higher than that under IC subsidy as *t*_0_ increases. Thus, the ER subsidy can better help manufacturers cope with a heavy carbon tax, and actively launch emissions reduction activities when the carbon tax is heavy. Then, for the government, ER subsidy should be adopted if a heavy carbon tax is levied; otherwise, IC subsidy is beneficial to manufacturers’ low-carbon production and their own development.

### 6.2. Effects of Consumer Low-Carbon Awareness

**Figure 2:**
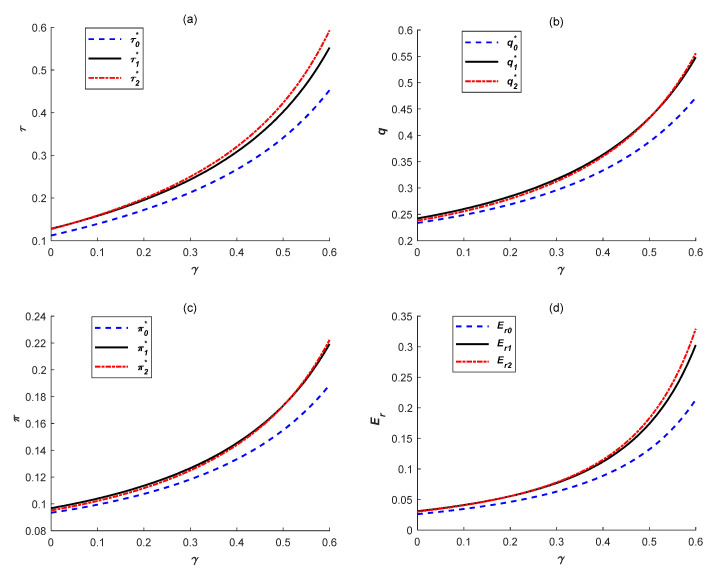
Effects of low-carbon sensitivity coefficient on (**a**) emissions reduction levels, (**b**) manufacturing quantities, (**c**) total profits and (**d**) emissions reduction quantities.

Similarly, to explore effects of low-carbon sensitivity coefficient on emissions reduction levels, manufacturing quantities, total profits and emissions reduction quantities in the above three cases, we set α=1,β=0.5, k=2.5,e=1,η=0.1. Additionally, the carbon tax rate was set as 1.2 (*t*_0_ = 1.2) and the results are shown in Figure 2.

As shown in Figure 2, all variable values are gradually increasing in the above three cases as low-carbon sensitivity coefficient *γ* increases. This indicates that enhancement of consumer low-carbon awareness can better help manufacturers achieve win–win situations of economic and environmental benefits. Thus, as mentioned above, it is significant for the government and manufacturers to positively propagandize low-carbon products, such as popularization and education of low-carbon environmental protection, diversification and vigorous publicity of low-carbon products. Moreover, all variable values are higher under both two government subsidies compared with that under no government subsidy, and the rising *γ* would also make the positive effect of government subsidies more apparent. Furthermore, as shown in Figure 2, each variable value under IC subsidy is gradually higher than that under ER subsidy as *γ* increases. Consequently, when more consumers accept and approve low-carbon products, the IC government subsidy can better encourage manufacturers to launch emissions reduction activities to achieve low-carbon production and their own development. This is mainly because, when consumers have stronger low-carbon awareness, manufacturers’ emissions reduction investment can be timely and fully repaid. Therefore, manufacturers can be effectively encouraged to improve emissions reduction investment.

### 6.3. Effects of Government Subsidies

**Figure 3:**
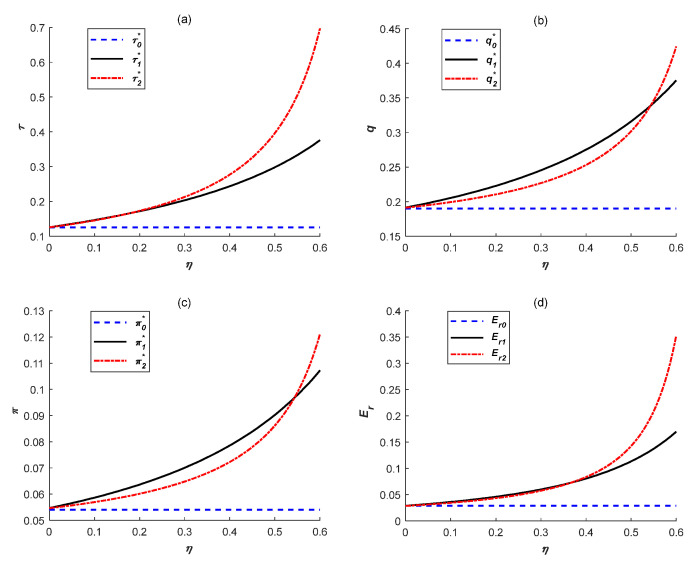
Effects of subsidy rate (**a**) emissions reduction levels, (**b**) manufacturing quantities, (**c**) total profits and (**d**) emissions reduction quantities.

**Figure 4:**
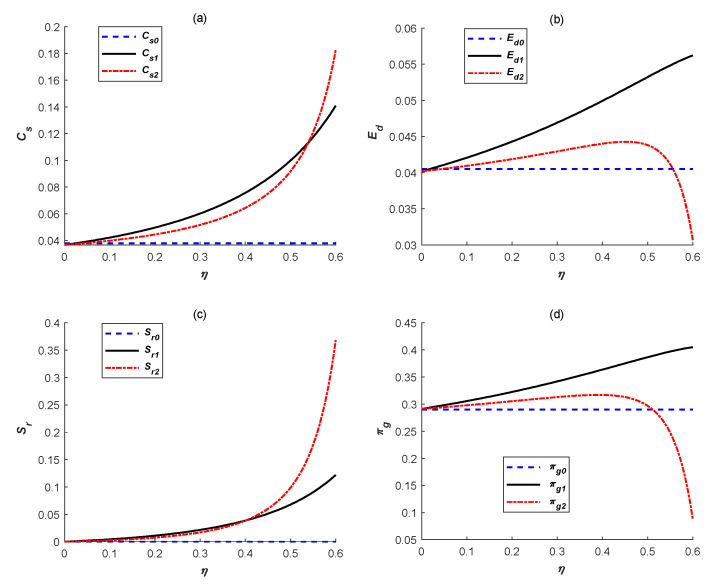
Effects of subsidy rate on (**a**) consume surplus, (**b**) environment damage, (**c**) amount of government subsidy and (**d**) social welfare.

To explore effects of subsidy rate on emissions reduction levels, manufacturing quantities, total profits, emissions reduction quantities and social welfare under the above two government subsidy policies, we set $\alpha =1,\beta =0.5,\;t_{0}=1.2,k=2.5,e=1.2,\gamma =0.1$. Additionally, the environmental damage coefficient was set as 0.2 (*μ* = 0.2) and the results are shown in Figure 3 and Figure 4.

As shown in Figure 3, all variable values are gradually increasing under ER subsidy and IC subsidy as subsidy rate *η* increases, which is consistent with Corollary 5. Meanwhile, each variable value under IC subsidy is gradually higher than that under ER subsidy as *η* increases. Therefore, for the government, it is necessary to determine which subsidy will be adopted considering the range of subsidy rate affected by government budget constraint. For instance, when the budget is enough and the government determines to raise subsidy rate, the IC subsidy is gradually more beneficial to manufacturers’ low-carbon production and their own development. In this way, the government could share manufacturers’ investment risk to some degree, which can effectively encourage manufacturers to improve emissions reduction levels.

Finally, we mainly analyze effects of government subsidy rate *η* on social welfare as shown in Figure 4. As the leader, the government needs to consider the manufacturer’s decision feedback and maximize social welfare by determining the specific subsidy policy. Similar to Yenipazarli [24], social welfare *π_g_* is defined as the sum of the manufacturer’s profit and consumer surplus minus the environmental damage cost. Among them, consumer surplus and environmental damage cost can be obtained by referring to Yenipazarli [24] and Cao et al. [51], respectively. Then, the social welfare function is shown as follows:

(4) $$\pi_{g}=(\frac{\alpha +\gamma \tau -q}{\beta }\cdot q-\frac{1}{2}k\tau^{2})+\frac{q^{2}}{2\beta }-\mu (1-\tau )eq$$

It can be observed from Figure 4d that social welfare under ER subsidy is always highest compared with that under no government subsidy and IC subsidy, and gradually increases as the rise of *η*. However, social welfare under IC subsidy initially increases and then decreases as *η* increases. Moreover, social welfare under IC subsidy begins to be lower than that under no government subsidy when *η* is higher than a certain critical value. The main reason is because increments of the manufacturer’s total profit almost come from the higher emissions reduction subsidies under IC subsidy as *η* increases. It can be easily found by comparing Figure 3c and Figure 4c. Thus, excluding emissions reduction subsidies, the manufacturer’s real profit (namely, sales revenue minus emissions reduction cost) is negative when subsidy rate is higher under IC subsidy. Thus, when *η* is higher than a certain critical value, although there is a higher consumer surplus and a lower environmental damage under IC subsidy, as shown in Figure 4a,b, social welfare is the lowest. In sum, ER subsidy can bring higher social welfare and have more obvious advantages.

## 7. Conclusions and Managerial Insights

To promote emissions reduction activities, the government provides emissions reduction subsidies under the carbon tax regulations. This paper considers a monopolistic manufacturer that jointly determines emissions reduction level and manufacturing quantity of low-carbon products under the government’s carbon tax regulation and ER/IC subsidy. The manufacturer’s optimal operational decisions are obtained in different cases by formulating three decision-making optimization models. Additionally, effects of carbon tax, consumer low-carbon awareness and emissions reduction cost coefficient are analyzed, and the subsidy effect of different government subsidies is emphatically investigated, resorting to some theoretical analysis and numerical analysis. Based on the analysis, the following conclusions and managerial insights can be drawn.

(1) A carbon tax regulation can always impel manufacturers to launch emissions reduction activities, which would bring the higher manufacturing quantities and total profits. However, whether there are government subsidies or not, a rising tax rate cannot always encourage manufacturers to further improve emissions reduction levels. When the carbon tax rate and emissions reduction cost coefficient are over the corresponding critical values, the higher carbon emissions cost and emissions reduction cost severely restrain manufacturers’ emission reduction and production activities. Therefore, for manufacturers, it is essential to independently develop or introduce efficient and low-cost emissions reduction technologies, which could make them more qualified to further improve emissions reduction investments. For the government, it is crucial to target raising or reducing the tax rate to further improve manufacturers’ emissions reduction levels, considering emissions reduction technology or investment cost. For instance, the government could levy a low carbon tax on manufacturers with underdeveloped emissions reduction technologies or higher emission reduction costs to properly promote emissions reduction and protect low-carbon production activities.

What is noticeable is that government subsidies could widen the conditions of carbon tax regulation in improving emissions reduction investments. Additionally, both ER subsidy and IC subsidy are always beneficial to manufacturers’ emissions reduction, outputs and their own benefits. Furthermore, compared with the case under no government subsidy, advantages of both two government subsidies would be more apparent in a heavy carbon tax circumstance. Consequently, for the government, a reasonable subsidy policy provided to manufacturers who launch emissions reduction activities is necessary, which not only brings some better results in low-carbon production and industrial development, but also helps manufacturers cope with a heavier carbon tax. In particular, for ER subsidy, it can induce the higher emissions reduction level, manufacturing quantity and profit compared with IC subsidy when the tax rate is higher. Therefore, the government should adopt the ER subsidy if a heavy carbon tax is levied; otherwise, the IC subsidy will be more advantageous.

(2) Stronger consumer low-carbon awareness can better help manufacturers achieve win–win situations of economic and environmental benefits, and make the positive effect of government subsidies more apparent. Thus, it is significant for the government and manufacturers to positively propagandize low-carbon products, such as popularization and education of low-carbon environmental protection, diversification and vigorous publicity of low-carbon products. Additionally, compared with ER subsidy, IC subsidy can induce higher emissions reduction levels, manufacturing quantity and profit when the low-carbon sensitivity coefficient is higher. Therefore, for the government, IC subsidy should be adopted in some industries where low-carbon products are widely accepted (e.g., household appliance industry), or in some regions where more local consumers have strong low-carbon awareness (e.g., economically developed areas). Otherwise, ER subsidy will be more advantageous.

(3) Besides diverse circumstances (e.g., carbon tax circumstance, industry circumstance or region circumstance), subsidies may also be selected by the government from different perspectives. For instance, from manufacturers’ and consumers’ perspectives, IC subsidy can bring better results when the subsidy rate is higher. Therefore, for the government, it is necessary to determine which subsidy will be adopted considering available government budget. When the budget is enough and the government is determined to raise the subsidy rate, the IC subsidy is gradually more advantageous. Additionally, if the government concentrates more on environment issues, IC subsidy will be a more effective policy to be adopted compared with ER subsidy. However, all things considered, ER subsidy can always bring higher social welfare and have more obvious advantages. Furthermore, IC subsidy is not always beneficial to social welfare. Specifically, when η is higher than a certain critical value, social welfare under IC subsidy begins to be lower than that under no government subsidy.

There are a few limitations in this study. First, in practice, the government always has a budget constraint, which should be considered to explore the effect of different subsidy policies. Additionally, there are often several product types in the market, such as green products, ordinary products and remanufactured products. Then, the competition among multiple products can be involved in future research. Another limitation of this study is that it only considers identical and fixed subsidy rates of different government policies, whereas this assumption can be relaxed to further explore the government’s decision-making process.

## Figures and Tables

**Table 1:** Relevant parameters and decision variables.

**Decision Variables**	
*q*	Manufacturing quantity of low-carbon products
*τ*	Emissions reduction level
**Relevant Parameters**	
*α*	Potential demand of low-carbon products
*β*	Product price sensitivity coefficient
*γ*	Low-carbon sensitivity coefficient
*p*	Sales price of unit low-carbon product
*e*	Emissions quantity of unit low-carbon product
*t*_0_	Carbon tax rate
*k*	Emissions reduction cost coefficient
*η*	Government subsidy rate
*μ*	Environmental damage coefficient
*π*	Manufacturer’s profit
*π_g_*	Social welfare
*E_r_*	Emissions reduction quantity
*E_d_*	Environmental damage cost
*C_s_*	Consumer surplus
*S_r_*	Amount of government subsidy

## Data Availability

The dataset used and/or analysed in this study is available from the corresponding author on reasonable request.

## Appendix A

**Proof** **of** **Proposition** **1.** According to the first derivation of total profit $\pi_{0}$ with respect to $\tau_{0}$ and $q_{0}$, we can obtain $\tau_{0}=\frac{(\gamma +\beta t_{0}e)q_{0}}{k\beta }$ and $q_{0}=\frac{\alpha -\beta t_{0}e+(\gamma +\beta t_{0}e)\tau_{0}}{2}$. Then, we have optimal emissions reduction level $\tau_{0}^{*}=\frac{\left(\alpha -\beta t_{0}e)(\gamma +\beta t_{0}e\right)}{2k\beta -{\left(\gamma +\beta t_{0}e\right)}^{2}}$ and manufacturing quantity $q_{0}^{*}=\frac{k\beta (\alpha -\beta t_{0}e)}{2k\beta -{\left(\gamma +\beta t_{0}e\right)}^{2}}$. Next, substituting $\tau_{0}^{*}$ and $q_{0}^{*}$ into Equation (1) and $E_{0}=eq_{0}(1-\tau_{0})$, the expression of $\pi_{0}^{*}$ and $E_{0}$ can be obtained. Moreover, because of α>βp and $p>t_{0}e$, we have $\alpha -\beta t_{0}e>0$. Furthermore, since $0<\tau_{0}^{*}<1$, we then have $2k\beta -{\left(\gamma +\beta t_{0}e\right)}^{2}>0$ and $2k\beta -(\alpha +\gamma )(\gamma +\beta t_{0}e)>0$. Meanwhile, $(\alpha +\gamma )(\gamma +\beta t_{0}e)>{\left(\gamma +\beta t_{0}e\right)}^{2}$ is always tenable, and thus above optimal solutions need to satisfy $2k\beta -(\alpha +\gamma )(\gamma +\beta t_{0}e)>0$. Therefore, Proposition 1 is proven. □

**Proof** **of** **Proposition** **2.** According to the first derivation of total profit $\pi_{1}$ with respect to $\tau_{1}$ and $q_{1}$, we can obtain $\tau_{1}=\frac{[\gamma +\beta t_{0}e(1+\eta )]q_{1}}{k\beta }$ and $q_{1}=\frac{\alpha -\beta t_{0}e+[\gamma +\beta te_{0}(1+\eta )]\tau_{1}}{2}$. Then, we have optimal emissions reduction level $\tau_{1}^{*}=\frac{\left(\alpha -\beta t_{0}e)[\gamma +\beta t_{0}e(1+\eta )\right]}{2k\beta -{\left[\gamma +\beta t_{0}e(1+\eta )\right]}^{2}}$ and manufacturing quantity $q_{1}^{*}=\frac{k\beta (\alpha -\beta t_{0}e)}{2k\beta -{\left[\gamma +\beta t_{0}e(1+\eta )\right]}^{2}}$. Next, substituting $\tau_{1}^{*}$ and $q_{1}^{*}$ into Equation (2) and $E_{1}=eq_{1}(1-\tau_{1})$, the expression of $\pi_{1}^{*}$ and $E_{1}$ can be obtained. In addition, since $0<\tau_{1}^{*}<1$, we then have $2k\beta -(\alpha +\gamma +\eta \beta t_{0}e)[\gamma +\beta t_{0}e(1+\eta )]>0$ and $2k\beta -{\left[\gamma +\beta t_{0}e(1+\eta )\right]}^{2}>0$. Furthermore, since $E_{1}>0$, we have $2k\beta -[\alpha (1+\eta )+\gamma ][\gamma +\beta t_{0}e(1+\eta ]>0$. Meanwhile, $[\alpha (1+\eta +\gamma )][\gamma +\beta t_{0}e(1+\eta )]>(\alpha +\gamma +\eta \beta t_{0}e)[\gamma +\beta t_{0}e(1+\eta )]>{\left[\gamma +\beta t_{0}e(1+\eta )\right]}^{2}$ is always tenable, and thus above optimal solutions need to satisfy $2k\beta -[\alpha (1+\eta )+\gamma ][\gamma +\beta t_{0}e(1+\eta )]>0$. Therefore, Proposition 2 is proven. □

**Proof** **of** **Proposition** **3.** According to the first derivation of total profit $\pi_{2}$ with respect to $\tau_{2}$ and $q_{2}$, we can obtain $\tau_{2}=\frac{(\gamma +\beta t_{0}e)q_{2}}{k\beta (1-\eta )}$ and $q_{2}=\frac{\alpha -\beta t_{0}e+(\gamma +\beta t_{0}e)\tau_{2}}{2}$. Then, we have the optimal emissions reduction level $\tau_{2}^{*}=\frac{\left(\alpha -\beta t_{0}e)(\gamma +\beta t_{0}e\right)}{2k\beta (1-\eta )-{\left(\gamma +\beta t_{0}e\right)}^{2}}$ and manufacturing quantity $q_{2}^{*}=\frac{k\beta (1-\eta )(\alpha -\beta t_{0}e)}{2k\beta (1-\eta )-{\left(\gamma +\beta t_{0}e\right)}^{2}}$. Next, substituting $\tau_{2}^{*}$ and $q_{2}^{*}$ into Equation (3) and $E_{2}=eq_{2}(1-\tau_{2})$, the expression of $\pi_{2}^{*}$ and $E_{2}$ can be obtained. Furthermore, since $0<\tau_{2}^{*}<1$, we then have $2k\beta (1-\eta )-{\left(\gamma +\beta t_{0}e\right)}^{2}>0$ and $2k\beta (1-\eta )-(\alpha +\gamma )(\gamma +\beta t_{0}e)>0$. Meanwhile, $(\alpha +\gamma )(\gamma +\beta t_{0}e)>{\left(\gamma +\beta t_{0}e\right)}^{2}$ is always tenable, and thus above optimal solutions need to satisfy $2k\beta (1-\eta )-(\alpha +\gamma )(\gamma +\beta t_{0}e)>0$. Therefore, Proposition 3 is proven. □

**Proof** **of** **Corollary** **1.** When the manufacturer does not conduct any emission reduction activity, the corresponding optimal manufacturing quantity, total profit and carbon emissions can be easily obtained: $q_{n}^{*}=\frac{\alpha -\beta t_{0}e}{2}$, $\pi_{n}^{*}=\frac{{\left(\alpha -\beta t_{0}e\right)}^{2}}{4\beta }$, $E_{n}=\frac{e(\alpha -\beta t_{0}e)}{2}$. Then, according to the expression of $q_{0}^{*}$, we can obtain $q_{0}^{*}-q_{n}^{*}=\frac{(\alpha -\beta t_{0}e){\left(\gamma +\beta t_{0}e\right)}^{2}}{4k\beta -2{\left(\gamma +\beta t_{0}e\right)}^{2}}>0$. Thus, $q_{0}^{*}>q_{n}^{*}$. Similarly, we can also obtain $q_{1}^{*}>q_{n}^{*}$ and $q_{2}^{*}>q_{n}^{*}$. Additionally, according to the expression of $\pi_{0}^{*}$, we can obtain $\pi_{0}^{*}-\pi_{n}^{*}=\frac{2{\left(\gamma +\beta t_{0}e\right)}^{2}{\left(\alpha -\beta t_{0}e\right)}^{2}}{4\beta [4k\beta -2{\left(\gamma +\beta t_{0}e\right)}^{2}]}>0$. Thus, $\pi_{0}^{*}>\pi_{n}^{*}$. Similarly, we can also obtain $\pi_{1}^{*}>\pi_{n}^{*}$ and $\pi_{2}^{*}>\pi_{n}^{*}$. Therefore, Corollary 1 is proven. □

**Proof** **of** **Corollary** **2.** Under no government subsidy, according to the expression of $\tau_{0}^{*}$, we can obtain $\frac{\partial \tau_{0}^{*}}{\partial t_{0}}=\frac{\beta e[(\alpha +\gamma ){\left(\gamma +\beta t_{0}e\right)}^{2}+2k\beta (\alpha -2\beta t_{0}e-\gamma )]}{{\left[2k\beta -{\left(\gamma +\beta t_{0}e\right)}^{2}\right]}^{2}}$. If $t_{0}<\frac{\alpha -\gamma }{2\beta e}$, then $\frac{\partial \tau_{0}^{*}}{\partial t_{0}}>0$. If $t_{0}>\frac{\alpha -\gamma }{2\beta e}$, only when $(\alpha +\gamma ){\left(\gamma +\beta t_{0}e\right)}^{2}+2k\beta (\alpha -2\beta t_{0}e-\gamma )>0$ (namely, $k<\frac{(\alpha +\gamma ){\left(\gamma +\beta t_{0}e\right)}^{2}}{2\beta (\gamma -\alpha +2\beta t_{0}e)}$), $\frac{\partial \tau_{0}^{*}}{\partial t_{0}}>0$; otherwise, $\frac{\partial \tau_{0}^{*}}{\partial t_{0}}<0$. Similarly, we can also obtain the other results. Therefore, Corollary 2 is proven. □

**Proof** **of** **Corollary** **3.** Under no government subsidy, according to the expression of $q_{0}^{*}$, we can obtain $\frac{\partial q_{0}^{*}}{\partial t_{0}}=\frac{k\beta^{2}e[(2\alpha +\gamma -\beta t_{0}e)(\gamma +\beta t_{0}e)-2k\beta ]}{{\left[2k\beta -{\left(\gamma +\beta t_{0}e\right)}^{2}\right]}^{2}}$. Then, $k<\frac{\left(2\alpha +\gamma -\beta t_{0}e)(\gamma +\beta t_{0}e\right)}{2\beta }\Leftrightarrow \frac{\partial q_{0}^{*}}{\partial t_{0}}>0$ and $k>\frac{\left(2\alpha +\gamma -\beta t_{0}e)(\gamma +\beta t_{0}e\right)}{2\beta }\Leftrightarrow \frac{\partial q_{0}^{*}}{\partial t_{0}}<0$. In addition, according to the expression of $\pi_{0}^{*}$, we can obtain $\frac{\partial \pi_{0}^{*}}{\partial t_{0}}=\frac{k\beta e(\alpha -\beta t_{0}e)[(\gamma +\beta t_{0}e)(\alpha +\gamma )-2k\beta ]}{{\left[2k\beta -{\left(\gamma +\beta t_{0}e\right)}^{2}\right]}^{2}}$. Since there is $k>\frac{\left(\gamma +\beta t_{0}e)(\alpha +\gamma \right)}{2\beta }$, thus $\frac{\partial \pi_{0}^{*}}{\partial t_{0}}<0$. Similarly, we can also obtain the other results. Therefore, Corollary 3 is proven. □

**Proof** **of** **Corollary** **4.** According to the expression of $\tau_{0}^{*}$, $q_{0}^{*}$ and $\pi_{0}^{*}$, we can obtain $\frac{\partial \tau_{0}^{*}}{\partial \gamma }=\frac{\left(\alpha -\beta t_{0}e)[2k\beta +{\left(\gamma +\beta t_{0}e\right)}^{2}\right]}{{\left[2k\beta -{\left(\gamma +\beta t_{0}e\right)}^{2}\right]}^{2}}>0$, $\frac{\partial \tau_{0}^{*}}{\partial k}=\frac{-2\beta (\alpha -\beta t_{0}e)(\gamma +\beta t_{0}e)}{{\left[2k\beta -{\left(\gamma +\beta t_{0}e\right)}^{2}\right]}^{2}}<0$, $\frac{\partial q_{0}^{*}}{\partial \gamma }=\frac{2k\beta (\alpha -\beta t_{0}e)(\gamma +\beta t_{0}e)}{{\left[2k\beta -{\left(\gamma +\beta t_{0}e\right)}^{2}\right]}^{2}}>0$, $\frac{\partial q_{0}^{*}}{\partial k}=-\frac{\beta (\alpha -\beta t_{0}e){\left(\gamma +\beta t_{0}e\right)}^{2}}{{\left[2k\beta -{\left(\gamma +\beta t_{0}e\right)}^{2}\right]}^{2}}<0$, $\frac{\partial \pi_{0}^{*}}{\partial \gamma }=\frac{k{\left(\alpha -\beta t_{0}e\right)}^{2}(\gamma +\beta t_{0}e)}{{\left[2k\beta -{\left(\gamma +\beta t_{0}e\right)}^{2}\right]}^{2}}>0$ and $\frac{\partial \pi_{0}^{*}}{\partial k}=-\frac{{\left(\alpha -\beta t_{0}e\right)}^{2}{\left(\gamma +\beta t_{0}e\right)}^{2}}{2{\left[2k\beta -{\left(\gamma +\beta t_{0}e\right)}^{2}\right]}^{2}}<0$. Similarly, we can also obtain the other results. Therefore, Corollary 4 is proven. □

**Proof** **of** **Corollary** **5.** According to the expression of $\tau_{1}^{*}$ and $\tau_{2}^{*}$, we can obtain $\frac{\partial \tau_{1}^{*}}{\partial \eta }=\frac{\beta t_{0}e(\alpha -\beta t_{0}e){\left\{2k\beta +{\left[\gamma +\beta t_{0}e(1+\eta )\right]}^{2}\right\}}^{2}}{{\left\{2k\beta -{\left[\gamma +\beta t_{0}e(1+\eta )\right]}^{2}\right\}}^{2}}>0$ and $\frac{\partial \tau_{2}^{*}}{\partial \eta }=\frac{2k\beta (\alpha -\beta t_{0}e)(\gamma +\beta t_{0}e)}{{\left[2k\beta (1-\eta )-{\left(\gamma +\beta t_{0}e\right)}^{2}\right]}^{2}}>0$. Similarly, $\frac{\partial q_{1}^{*}}{\partial \eta }=\frac{2k\beta^{2}t_{0}e(\alpha -\beta t_{0}e)[\gamma +\beta t_{0}e(1+\eta )]}{{\left\{2k\beta -{\left[\gamma +\beta t_{0}e(1+\eta )\right]}^{2}\right\}}^{2}}>0$, $\frac{\partial q_{2}^{*}}{\partial \eta }=\frac{k\beta (\alpha -\beta t_{0}e){\left(\gamma +\beta t_{0}e\right)}^{2}}{{\left[2k\beta (1-\eta )-{\left(\gamma +\beta t_{0}e\right)}^{2}\right]}^{2}}>0$ and $\frac{\partial \pi_{1}^{*}}{\partial \eta }=\frac{k\beta t_{0}e{\left(\alpha -\beta t_{0}e\right)}^{2}[\gamma +\beta t_{0}e(1+\eta )]}{{\left\{2k\beta -{\left[\gamma +\beta t_{0}e(1+\eta )\right]}^{2}\right\}}^{2}}>0$, $\frac{\partial \pi_{2}^{*}}{\partial \eta }=\frac{k{\left(\alpha -\beta t_{0}e\right)}^{2}{\left(\gamma +\beta t_{0}e\right)}^{2}}{2{\left[2k\beta (1-\eta )-{\left(\gamma +\beta t_{0}e\right)}^{2}\right]}^{2}}>0$. Then, $\tau_{1}^{*}>\tau_{0}^{*}$, $\tau_{2}^{*}>\tau_{0}^{*}$, $q_{1}^{*}>q_{0}^{*}$, $q_{2}^{*}>q_{0}^{*}$ and $\pi_{1}^{*}>\pi_{0}^{*}$, $\pi_{2}^{*}>\pi_{0}^{*}$. Therefore, Corollary 5 is proven. □

**Proof** **of** **Corollary** **6.** According to expression of $\tau_{1}^{*}$ and $\tau_{2}^{*}$, we can obtain $\tau_{1}^{*}-\tau_{2}^{*}=\frac{\beta \eta (\alpha -\beta t_{0}e)\{t_{0}e(\gamma +\beta t_{0}e)[\gamma +\beta t_{0}e(1+\eta )]-2k(\gamma +\eta \beta t_{0}e)\}}{\left\{2k\beta -{\left[\gamma +\beta t_{0}e(1+\eta )\right]}^{2}\}\{2k\beta (1-\eta )-{\left(\gamma +\beta t_{0}e\right)}^{2}\right\}}$. Therefore, Corollary 6 can be proven. □

**Proof** **of** **Corollary** **7.** According to expression of $q_{1}^{*}$ and $q_{2}^{*}$, we can obtain $q_{1}^{*}-q_{2}^{*}=\frac{k\beta (\alpha -\beta t_{0}e)\{(1-\eta ){\left[\gamma +\beta t_{0}e(1+\eta )\right]}^{2}-{\left(\gamma +\beta t_{0}e\right)}^{2}\}}{\left\{2k\beta -{\left[\gamma +\beta t_{0}e(1+\eta )\right]}^{2}\}[2k\beta (1-\eta )-{\left(\gamma +\beta t_{0}e\right)}^{2}\right]}$. Additionally, according to expression of $\pi_{1}^{*}$ and $\pi_{2}^{*}$, we have $\pi_{1}^{*}-\pi_{2}^{*}=\frac{k{\left(\alpha -\beta t_{0}e\right)}^{2}\{(1-\eta ){\left[\gamma +\beta t_{0}e(1+\eta )\right]}^{2}-{\left(\gamma +\beta t_{0}e\right)}^{2}\}}{2\{2k\beta -{\left[\gamma +\beta t_{0}e(1+\eta )\right]}^{2}\}[2k\beta (1-\eta )-{\left(\gamma +\beta t_{0}e\right)}^{2}]}$. Therefore, Corollary 7 can be proven. □
